# Supplementary material for: Are carbon emissions trading and green financial instruments synergistic? -Comprehensive quantitative research based on content analysis
Source: PLoS One. 2024 Mar 7;19(3):e0298601. doi: 10.1371/journal.pone.0298601 (PMC10919601; doi:10.1371/journal.pone.0298601)
Supplement: S1 Data — (ZIP) [file pone.0298601.s001.zip › Data/地级市Z_F工作报告.zip!/─■╧─/╓╨╬└╩╨/└┤╫╘╣1⁄2╓┌║┼í╛┬φ┐╦╩2╛▌═°í┐_2015.docx]

各位代表：

我代表市人民政府，向大会作工作报告，请予审议，并请政协委员和列席人员提出意见。

2014年工作回顾

2014年是中卫建市十周年，也是开启二次创业新征程之年。面对经济持续下行压力和产业转型升级重任，我们知难而进，攻坚克难，主动作为，依法履职，较好地完成了市三届人大二次会议确定的主要任务。实现地区生产总值296.9亿元，增长5.4%；地方公共财政预算收入19.5亿元，增长14%；固定资产投资334亿元，增长5.5%；社会消费品零售总额56.7亿元，增长12.4%；城镇居民人均可支配收入19931元，增长8.2%；农村常住居民人均可支配收入7403元，增长10.8%。

一年来，重点抓了六方面工作：

一、深化改革创新，发展环境进一步优化

扩大对外开放。全面深化与港中旅合作，推动沙坡头景区公司向混合所有制和集团化发展。采用“前店后厂”区域合作模式创建宁夏中关村科技产业园，美国亚马逊AWS等世界云计算龙头企业落户中卫。多渠道、宽领域开展选商引资，与中国移动、协鑫集团等56家企业签订投资合作协议，招商引资区外到位资金249.6亿元。积极与北京、江苏、重庆等发达地区及周边地区产业对接，构筑全方位、多层次对外开放新格局。

创新体制机制。全面构建开放型经济，深化生态文明体制，健全城乡发展一体化体制机制，推进文化体制机制等改革创新。成立沙坡头旅游经济开发试验区管委会，搭建旅游产业化发展新格局。设立海兴开发区，探索海原扶贫开发新模式。创新投融资体制，发行7亿元旅游企业债券，吸引宽带资本、协鑫集团设立股权投资基金。成功引进淘宝网、渤海商品交易所等电商平台。成立宁夏枸杞产业发展联盟。推行“计量到户、计费到户”农业综合水价改革，实施农村土地确权登记，沙坡头区开展农村产权流转试点。

优化“两优”环境。加快推进行政审批制度改革，出台《中卫市“两优”行政审批制度》，推行负面清单审批模式，审批主体由分散变集中，由多家审批变一家受理，审批事项由229项压减到80项，减少65%。市本级行政“零收费”，为企业减负1880万元。实行“先证后照”，简化审批环节，释放市场活力，新登记注册企业2489家，增长86%。设立总受理窗口，所有行政审批事项前台集中受理、后台并联审批、事后加强监管、一站限时5天办结，共办结审批事项12.8万件，现场办结率87.5%，办件及时率100%，群众满意率99.5％。

二、加快产业转型，结构调整取得明显成效

旅游产业实现新突破。完善吸引物系统，推出旋翼机、房车、沙漠探险等新产品，开发银阳光伏科技生态园、金沙岛景观提升、南长滩党项民俗村等一批独具特色的旅游景点。完善综合配套服务系统，推进沙坡头水镇和秀水岛等旅游主题功能区建设。完善交通组织系统，组织9个重点客源城市包机89架次、专列32列次。启动旅游标准化建设和智慧旅游工程，不断提升旅游核心竞争力，沙坡头景区游客井喷式增长，十一爆满。全年接待游客342万人次，实现旅游收入26.4亿元，分别增长31.2%和24.9%。中卫被评为全国旅游标准化示范城市。

信息产业迈出新步伐。加快推进宁夏中关村科技产业园及西部云基地规划建设，云创公司为中国电信、奇虎360量身定制的数据中心主体工程已完工，西部云基地为亚马逊代建的新一代数据中心开工建设。中国移动数据中心签订投资协议，中国联通数据中心完成可研报告。编制中阿互联网经济试验区（网上丝绸之路）规划方案，启动中航信集团等央企灾备中心建设，国家战略数据安全储备基地项目前期工作顺利推进。北京与中卫“前店后厂”骨干网络通道系统完成测试。积极与华为、浪潮、美国ZT、戴尔等国内外云计算企业对接，项目洽谈进展顺利。实施光纤到户工程，市区光网实现全覆盖。

现代物流业开创新局面。加强交通物流基础设施建设，启动实施中卫铁路“3213”规划，干武铁路开工建设，吴忠至中宁城际铁路、太中银铁路增建二线等项目前期工作加快推进；中卫、中宁黄河大桥竣工通车，海原至三河快速通道进展顺利。成功引进中国物流集团、中陕物流集团建设中卫、中宁两个现代综合物流园。中卫西部货物快运中心启动运营，开通中卫至玉门等3条货物快运环线。中宁陆路口岸与银川综合保税区及天津港联营合作深入推进。甘塘被国家发改委纳入全国30个煤炭物流园区建设规划，中卫交通及物流产业迎来历史性机遇。

现代农业取得新成效。实施农业振兴计划，建成设施蔬菜标准化生产基地20万亩，提质增效示范园8个，试验示范瓜菜新品种30个。新增枸杞2.8万亩，建成优质硒砂瓜品质品牌保护区40万亩。完成玉米、蔬菜、花卉制种5.3万亩。新建千头奶（肉）牛养殖场23个，奶牛存栏4.7万头、肉牛饲养量27.5万头，分别增长34.3%、14.6%。建成10万亩高效节水农业示范基地，亩均节水560方，节水率65%。流转土地41.5万亩，464家新型农业经营主体参与农业标准化生产。实施农业加工项目34个，农产品加工产值增长35.3%。粮食生产创历史新高，实现农业增加值47.6亿元，增长5.8%，一改近年来停滞不前的局面。

新型工业取得新进展。围绕新能源、新材料、精细化工三大产业，开工建设中电投2×35万千瓦热电联产、兴尔泰120万吨硝基复合肥循环经济等18个重大项目，众磊鑫180万吨钒钛合金、重庆紫光一期5万吨蛋氨酸等行业领先项目建成投产。启动国家级甘塘新能源产业示范区规划编制，光伏、风电装机容量分别达1190兆瓦和1680兆瓦。针对工业下行和企业生产经营困难，建立市级领导“三个十”包抓责任制，向困难企业派驻解困工作组，落实电力直接交易、差别化电价、路桥通行费减征等扶持措施，协调解决天元锰业融资、土地和项目环评等困难，帮助企业渡过难关。规模以上工业企业达115家，实现增加值89.8亿元，增长2.7%。

三、统筹城乡发展，人居环境持续改善

倾心打造城市。坚持以绿为美、以水为源、以净为荣、以适为宜、以人为本，实施沿黄景观带建设和城市街巷畅通、亮化、美化“四大工程”，建成十里水街和“大河之舞”主题文化公园。完成30个老旧小区91万平方米基础设施、供热计量及外墙保温工程，改造城市棚户区5155户。开工建设南苑西路延伸段等8条城市道路，完成西二道巷等3条巷道改造，城市基础设施日益完善。实施新老城区绿化改造工程，栽植乔灌木430万株、水生植物13万株，城市绿地率和绿化覆盖率分别达36.6%和37.9%。老城区城中村及棚户区改造项目荣获中国人居环境范例奖。

精心管理城市。出台《中卫市市容秩序管理办法》，整治随意摆摊和违法占道经营行为，实现市容秩序管理标准化、长效化。启动市区静态交通管理，建成智能停车场118个、人工停车场20个，划定泊车位4360个。加强小区物业管理，提升服务水平。创新城市管理模式，完善“以克论净”深度保洁机制，城市保洁自觉机制全面建立，赢得国家住建部充分肯定，中卫成功创建国家园林城市和自治区卫生城市。

用心建设新农村。编制完成《村镇布局规划》及东园等5个镇总体规划和57个中心村建设规划。投资1.7亿元，开工建设沙坡头区迎水桥镇、中宁县石空镇、海原县李旺镇等8个特色小城镇和敬农村等19个美丽村庄，改造农村危房6161户，建成农村公路395公里。深入实施以乡村主干道路为主的农村环境大整治，拆除违章建筑和破旧房屋6671户123万平方米，统一设置乡村道路标志线和信号灯等，配套完善 “户分类、村收集、镇转运、市县处理”的垃圾清运系统，农村人居环境明显改善。

四、持续改善民生，社会更加和谐稳定

办好民生实事。新增财力70%、压减公用经费5%全部用于改善民生。市区公交全覆盖、中小学冬季取暖等15件民生实事全部落实。宁夏大学中卫校区建设进展顺利，当年招生355人。扎实推进义务教育均衡发展，沙坡头区、中宁县通过自治区评估验收。在全区率先开展科技型中小企业风险补偿专项资金试点，获得首批6000万元授信贷款。实施沙漠光伏生态旅游科技试验示范项目，建成光伏生态大棚8座。深入推进文化惠民工程，完成黄河宫布展，建成杞乡黄河体育中心和海原新图书馆。开展文化下乡960场次，完成农村数字电影放映7443场。成功举办环青海湖国际公路自行车赛中卫赛段、全国大漠健身运动大赛等活动。组建新闻传媒中心。深入开展核心价值观“六进”活动，实施民风建设“五大工程”，创建全国文明单位（村镇）7个，评选表彰“最美中卫人”15名，重奖了舍己救人的贵州籍农民工徐传红等一批道德楷模，中卫顺利通过自治区文明城市验收。建成市人民医院医疗中心和186所标准化村卫生室。实施“单独两孩”生育政策，人口出生率控制在12.48‰以内。

完善保障体系。实行公租房、廉租房并轨管理，建成各类保障性住房1.63万套。坚持以创业带动就业，创办小企业460家，新增就业岗位4280个，转移农村劳动力15.8万人，城镇登记失业率控制在3.93%。加强养老服务体系建设，新扩建敬老院3所，新增床位370张。实施社会保险和公积金参保扩面工程，推行医疗费用医保联网即时结算制度。大病医保、城乡低保、五保供养、困难救助、重度残疾人津贴等“兜底”政策全面落实。中卫被确定为全国七星级慈善城市和全国社保卡综合应用试点示范城市。

实施精准扶贫。深入推进百万贫困人口扶贫攻坚战略，整合资金3.3亿元，实施85个重点贫困村整村推进工程，17个贫困村基本脱贫，减少贫困人口2.3万人。建成生态移民住房1422套，搬迁安置1012户4451人。有效解决沙坡头移民区生产生活突出问题，改良生沙荒地3万亩，入栏基础母羊4万只。协调华润集团加大海原帮扶力度，开工建设华润草畜一体化、希望小镇、经济生态林等项目。为海兴开发区量身定制19条优惠政策，促进经济社会快速发展。

创新社会治理。扎实推进平安中卫建设，进一步完善立体化社会治安防控体系，深入开展“禁毒人民战争”“六项整治”等专项行动，群众安全感不断增强。实行社区网格化管理，创新建立医疗和交通事故赔偿纠纷第三方调处机制和信访工作“1+X”制度体系，实行市级领导包抓重点信访案件和轮班接访制度，信访人次同比下降42%。实行餐饮服务食品安全无遗漏监管，建成安全生产在线智能视频监控系统和安全隐患自查自报系统，排查整改隐患6680条，整改率98%，安全生产形势总体平稳。全面落实民族宗教政策，依法管理宗教事务，狠抓宗教场所安全整治和宗教领域矛盾纠纷排查调处，创建国家级民族团结进步模范单位2个、自治区级7个，成为民族团结靓丽名片。

五、加强环境保护，美丽中卫建设加快推进

实施生态修复工程。扎实推进节水型社会建设，实施沙坡头南北干渠及灌区节水改造、二干渠改线、农村安全饮水工程等重点项目建设。引入民间投资2.2亿元建设峡门水库，开创了民间资本进入重大水利工程建设的先例。实施腾格里沙漠防沙治沙、南华山水源涵养林、移民迁出区生态修复、天然林资源保护、农田林网、城乡绿化等重点生态工程，完成造林28.3万亩，生态环境进一步改善。南华山晋升为国家级自然保护区。

加大环境保护力度。严格工业项目环境准入标准，实施工业园区节能环保行动计划，建立环境质量量化控制体系，对19家企业实施环境在线监测。成立中卫、中宁工业园区环保分局，加强环境监管。依法科学处置明盛染化环境污染敏感问题，果断拆除厂房设备、截断污染源、实施污染检测、安全处理废水、科学处置固废、严格追责问责。同时，举一反三，对154家工业企业开展拉网式环境风险隐患大排查，责令47家企业整改。

狠抓节能减排降耗。全面开展用煤企业能源审计，对冶金等重点行业实行对标管理。用新技术改造传统工业，实施新华冶炼尾气余热利用等6个节能技改项目，淘汰落后产能生产线8条，削减能源消费量52万吨标煤，万元GDP综合能耗下降9.8%。优化结构减排、强化工程减排、狠抓管理减排，化学需氧量、氨氮、二氧化硫、氮氧化物排放量4项约束性指标同比分别减少5.7%、24.5%、4.7%、17.5%，超额完成自治区下达任务。

六、切实转变职能，政府效能不断提升

加快法治政府建设。出台《中卫市重大行政决策规则》，确保政府决策公开、透明。自觉接受人大法律监督和政协民主监督，办理人大代表建议49件，当年办结率81.6%，答复率100%；政协委员提案160件，当年办结率92.5%，答复率100%。积极支持民主党派、工商联、无党派人士参政议政，密切联系工会、共青团、妇联等人民团体。扎实开展“六五”普法，实施公务员法律培训考试五年规划，培训1.95万人次。坚持依法行政，建立法律顾问制度，与市财政全额拨款单位签订顾问合同160份。积极开展行政复议委员会试点，受理案件102件，全区现场会在中卫召开。

加强政府廉政建设。大力开展“清廉中卫”建设，认真落实“一岗双责”，强化主体责任意识，严格执行 “一把手”五不直接分管制度。严格贯彻落实中央“八项规定”，深入开展工程建设领域突出问题及机关效能建设专项治理，停建各类楼堂馆所，清理腾退办公用房4.2万平方米，会议、文件分别同比减少35.7%和33%，“三公”经费同比压缩22.5%。规范政府投资项目招标管理，推行预算控制价、标书制作人、招标人、项目建设单位“四分离”。实行公共资源集中阳光交易，节约资金2.3亿元。加强国有资产管理，加大审计监督力度，坚持“零容忍”惩治腐败，查办违纪违法案件146件，给予党政纪处分139人，收缴违纪资金395万元。

强化政府效能建设。扎实开展党的群众路线教育实践活动，市政府领导班子整改“四风”方面突出问题39项。召开市政府常务会议27次，督办重点工作61项，研究决定重大事项189项。扎实开展“转作风、抓发展、大干150天”活动，建立市政府班子成员干事档案，公开政府领导工作周志，自觉接受社会监督。实行广场和媒体等多元化问政，通报了市环境保护局等3个单位不作为、慢作为、乱作为典型问题，对工作落实不力、作风散漫的51名责任人进行了问责。全面完成政府机构改革，撤并11个部门和事业单位，新组建5个，政府职能进一步转变。市长信箱受理解决群众诉求3175件，答复办结率100%。2014年全区群众评议机关和干部作风中卫列五市第一。

全民国防教育、双拥共建、民兵预备役、防震减灾、人民防空、外事侨务、统计调查、市志档案、邮政通信、气象服务等各项事业取得新成绩。

各位代表，过去一年，受外部环境和经济下行影响，加之我市部分行业产能过剩，房地产市场下滑等多种因素叠加，我市实现5.4%的经济增速，虽然低于10%的年初预期目标，但在转型升级、节能降耗、改善民生、改革创新等方面取得重大进展，为长远可持续发展打下了基础、汇集了能量，成绩难能可贵，实属不易！这是自治区党委、政府亲切关怀、大力支持的结果，是市委总揽全局、正确领导的结果，是市人大、政协支持配合、有效监督的结果，是全市广大干部群众真抓实干、共同努力的结果，是社会各界凝心聚力、和衷共济的结果。在此，我代表市人民政府，向全市人民，向人大代表、政协委员，向各民主党派、工商联、无党派人士、人民团体，向驻卫部队、武警官兵，公安干警，向所有关心、支持和参与中卫发展的各界人士、企业家，表示崇高的敬意和衷心的感谢！

过去一年，我市改革发展取得了一定成绩，但也存在一些不容忽视的问题，主要表现在：经济持续健康发展的基础不牢，制约发展的瓶颈还没有有效解决；转型升级压力加大，工业结构不尽合理，倚重倚能局面尚未根本改变，企业面临困难较大，战略性新兴产业有待培育；山川发展不平衡，扶贫攻坚任务艰巨，生态移民还存在一些后续问题，实现全面小康社会目标任重而紧迫；发展中对环境保护有不到位的地方，监管还存在漏洞，环保任务依然艰巨；全社会法治信仰和法律素养还不够高，司法不公和违法行政问题仍时有发生，信访矛盾纠纷在一些地方和领域比较突出；作风建设成果还不够稳固，少数干部为政不为、作风不实的现象依然存在。我们将高度重视，对症施策，切实加以改进。

2015年主要工作

今年是转型升级、二次创业的关键之年，是全面推进依法治市的开局之年，也是全面完成“十二五”规划的收官之年。政府工作的总体要求是：坚持以科学发展观为统领，认真贯彻落实党的十八大和十八届三中、四中全会、自治区党委十一届五次全会、自治区“两会”及市委三届五次全体会议精神，坚持稳中求进工作总基调，主动适应经济发展新常态，以提高经济发展质量和效益为中心，以改革开放为动力，以“四主一化”产业转型升级为主攻方向，以重大项目建设为载体，以改善民生为根本目的，坚持科学发展、创新驱动、依法治市，组织实施好“1746”工作计划，加快建设开放中卫、富裕中卫、和谐中卫、美丽中卫。

主要预期目标：地区生产总值增长8%，地方公共财政预算收入增长9%，固定资产投资增长15%以上，社会消费品零售总额增长11%，城镇居民人均可支配收入增长9%，农村常住居民人均可支配收入增长10%，城镇登记失业率控制在4.5%以内，万元GDP综合能耗下降7.7%，完成自治区下达的节能减排任务。

按照市委工作部署，重点抓好六方面工作：

一、积极主动适应新常态，推动经济转型升级

准确把握全国经济发展新常态，围绕自治区对中卫产业发展定位，大力实施“四主一化”的产业转型升级行动计划，构建现代化产业体系。

优先发展旅游业。高起点编制旅游产业规划，调整沙坡头国家级自然保护区范围和功能区，加快旅游经济开发试验区和旅游新镇核心区建设。实施沙坡头景区提升改造、腾格里沙漠湿地国家级旅游度假区、沙坡头水镇、银阳光伏科技生态园等重点工程，提升旅游核心竞争力。推进沙漠博物馆、高墩湖养老养生产业园、中宁中国枸杞文化产业园和海原天都山石窟等旅游开发项目，打造枸杞生态养生、黄河漂流、沙漠探险、湿地休闲、回乡风情、登山攀岩等差异化旅游产品。开通“沙坡头号”豪华旅游专列，构建西部特色原生态旅游通道。建设智慧旅游数据库和旅游平台，促进旅游与城市、旅游与文化深度融合发展，建设国际影响力的旅游目的地，力争接待游客人数和旅游总收入均增长25%以上。

积极发展信息产业。加快建设宁夏中关村科技产业园及西部云基地，完善基础设施、落实配套政策。启动运营亚马逊AWS、云创公司2个新一代数据中心；开工建设中国移动、中国联通、央企灾备基地等数据中心；争取微软、华为等数据中心项目签约落地，力争服务器规模达到20万台。加快中卫—太原—北京、中卫—西安—北京长途网络通道和2个110KV变电站等数据中心配套项目建设。加速4G网络进程，扩大信息消费。启动“智慧产业城”项目，推进与美国ZT、浪潮等配套龙头企业务实合作。立足中关村西部云基地，依托中阿合作平台，开创网上丝绸之路，为“一带一路”开疆扩土、独辟蹊径，着力构建云设施、云应用、云服务核心产业链，打造国家战略数据安全储备基地。

加快发展现代物流业。加快实施铁路“3213”规划，开工建设货物快运中心、物流中心、客运中心，构筑快速便捷物流通道。加快建设干武铁路增建二线，推进包兰铁路扩能改造及中卫火车站迁建、吴忠至中宁城际铁路、乌玛高速中卫段、海原至三河快速通道、迎闫公路和广申大道跨铁立交桥等项目建设。加快中国物流中卫物流园建设，开工建设甘塘煤炭物流园和宁夏星火国际物流园。大力发展通用航空，不断拓展新航线。扎实推进中宁陆路口岸与天津港、银川综合保税区跨区域合作。大力发展电子商务、供应链物流、金融物流等，打造区域现代物流中心。充分发挥欧亚大陆桥和丝绸之路经济带交通区位优势，加快构筑中阿陆上丝绸之路区域枢纽。

大力发展现代农业。以农业振兴计划为引领，坚持走“一特三高”发展路子，做优做强枸杞、硒砂瓜、蔬果、马铃薯和草畜等特色产业。新增枸杞5万亩，建成国家级绿色出口枸杞基地2万亩。完成高效节水农业5万亩，着力打造特色农业示范基地。规划都市工业园，重点扶持万齐、早康、夏华等一批重点农副产品加工企业发展。鼓励发展家庭农场、涉农网商、专业合作社等新型农业经营主体，培育市级示范合作社15家、家庭农场5家。加快培育壮大四季鲜、枸杞交易城等大型综合农产品批发市场，完善信息平台、冷链系统、质量追溯和加工配送等设施，不断提升农业现代化水平，力争农业增加值增长5%以上。

着力推动新型工业化。大力发展新能源产业，加快甘塘新能源产业基地建设，重点推进单晶硅、多晶硅、电池组件、风机制造、钢构制造等产业链项目，实施分布式光伏发电项目示范与应用，打造中卫新能源示范区。积极发展新材料产业，实施国家级循环化改造示范试点项目，加快中宁新材料基地建设，重点发展高端铝材、铝板带箔、铝合金等新材料。科学发展生物医药和精细化工产业，重点推进重庆紫光二期5万吨蛋氨酸、鑫华威30万吨炼焦油、利安隆3000吨高分子抗氧化剂、苏化集团张家港基地迁移等项目建设。加快宁钢与酒钢合作，实现资源、资本、资金的优化配置。出台中卫市企业科技创新后补助实施办法，加大科技自主创新和集成创新，全年专利申请量达到100项、授权量60项。力争规模以上工业增加值增长11%以上。

二、加快推进项目建设，增强经济发展后劲

依托《宁夏空间发展战略规划》，科学编制“十三五”规划，精心谋划事关中卫发展的重大项目，以项目扩投资、促发展、实现新跨越。

科学谋划重大项目。抢抓“一带一路”“两带两轴”、内陆开放型经济试验区建设、中央民族工作会议、自治区60大庆等重大机遇，科学编制好“十三五”经济社会发展规划，高质量编制完成16个专项规划。争取国家环保部对沙坡头国家级自然保护区核心区和缓冲区进行调整，加快推进沙坡头旅游经济开发试验区项目建设。启动中阿互联网经济试验区和网上丝绸之路项目前期工作，落实《国务院关于促进云计算创新发展培育信息产业新业态的意见》，将西部云基地纳入国家云计算产业重点布局规划。

加快实施重点项目。实施包兰铁路扩能改造、新一代数据中心、新能源发电等40个重点开工项目，争取开工率达95%，完成投资300亿元。加快推进重庆长寿化工氯丁橡胶、中化国际20万吨聚氨酯系列产品、协鑫集团超高效单晶、太中银铁路复线等16个预开工项目。扎实推进大柳树水利枢纽、甘塘新能源产业示范基地等10个重大项目前期工作，力促取得突破性进展。

积极引进招商项目。健全完善市级领导定点包抓重大招商项目、招商项目准入评估、招商引资联席会议等制度，形成市级领导顶层推动、主管部门牵头负责、县（区）部门协同推进的招商引资新机制。继续开展 “6312”专题招商，修订完善优惠政策，紧盯新兴产业引进、传统产业升级、优势产业扩规，多联产耦合延伸，以增量保持经济增长，以增量稀释重工业，进一步优化产业结构。以丝绸之路交通枢纽和中卫中关村科技园为平台，吸引外资和项目。严格项目准入机制，变“招商引资”为“选商引资”。积极对接，力促江苏悦达集团、天士力集团枸杞种植及深加工等55个洽谈项目落地，年内完成招商引资到位资金300亿元。

三、切实保障和改善民生，提升人民幸福指数

民生连着民心，民心凝聚民力。从一件件具体事情做起，从一个个具体问题抓起，让人民群众共享改革发展成果。

大力发展社会事业。宁夏大学中卫校区建成投入使用，积极筹建中阿科技大学。实施贫困家庭高中阶段免费教育，年内高中阶段毛入学率达到90%以上。改造东台学校等13个薄弱学校，完成黑林学校等7个体育场建设。沙坡头区和中宁县义务教育均衡发展工作力争通过国家验收。继续深化文化体制改革，完善四级公共文化服务体系，加大文艺精品创作扶持力度，促进文化产业发展。积极申报国家级和自治区级非遗重点名录项目，加大非物质文化遗产传承保护。开展“文化下乡”惠民演出800场、大型广场文艺演出100场，新建乡镇文化中心6个，村级健身广场14个，放映农村数字电影7000场次。实行体育场馆分时段对市民开放，最大限度满足市民健身需求。统筹规划建设医疗卫生设施，开工建设市疾病预防控制中心大楼。实行免费孕前优生健康检查，提高出生人口素质，人口出生率控制在14.37‰以内。

实施扶贫攻坚行动计划。全面完成“十二五”生态移民搬迁任务，扎实推进“四到”扶贫工程，坚持精准扶贫、整村推进，年内完成30个重点贫困村脱贫销号任务。对建档立卡贫困人口分类制定帮扶措施，确保项目进村到户。加大贫困人口转移培训力度，采取企业“订单式”培训，财政补贴与企业就业挂钩，实现户均有1名懂技术有特长的劳动力。做好驻村帮扶，拓宽社会帮扶，加强闽宁协作、华润集团定点帮扶，以示范村、示范户、专业合作社带动扶贫产业发展，引导帮助贫困地区走“造血式”发展路子，年内减少贫困人口2.3万人。

实施民生改善行动计划。办好12件民生实事。启动西关新村等集中片区2220户棚户区改造工程，开工建设各类保障性住房1.05万套。实施老建筑外墙保温改造120万平方米，改造老旧小区46个，引入专业公司规范小区物业管理。建设农村老年幸福院56个，积极鼓励社会资金投向养老产业，构建“9073”养老服务新模式。加强公积金归集、扩面工作。沙坡头区城乡居民基础养老金标准由100元提高到115元。实施农村安全饮水工程，用两年时间，解决海原县11个乡镇21.8万人饮水问题。积极拓宽创业就业渠道，农村劳动力转移就业15万人。建设“六位一体”的法律援助工作机制和以三级分类实施的法律服务全覆盖体系。

创新社会治理体系。继续深化“平安中卫”建设，严厉打击暴力犯罪，集中开展禁毒、打击侵财等专项行动，提升人民群众安全感。坚持安全生产党政同责、“五个全覆盖”，及时排除安全生产隐患，推行第三方监管机制，确保不发生重特大安全生产事故。加强市场监管，整合监管力量，下移监管重心，推行“网格化”监管和综合化执法，营造健康安全市场环境。加强气象、地震等预测预报，提高突发事件的应急处置能力，最大限度保障人民群众生命财产安全。进一步完善信访工作“1+X”制度体系，确保信访秩序持续明显好转。继续深化民族团结进步创建活动，承办好甘肃、宁夏两省区第九次宗教工作联席会。依法加强宗教事务管理，确保民族和睦、宗教和顺、社会和谐。

四、扎实推进生态文明建设，全力打造美丽中卫

全面落实《宁夏空间发展战略规划》，按照副中心城市定位，突出“沙漠水城、花儿杞乡、休闲中卫”城市特色，以沙坡头区为核心，以中宁县和海原县为两翼，提升城市内涵品质，强化城市功能，提升城市核心竞争力。

让城市更加宜居。精心筹备好全国城市建设管理工作现场会，全面展示中卫城市建设管理亮点和经验。积极推进红星美凯龙家居广场、悦达城市商贸综合体、城市文化广场等项目，切实提升城市品位。新建、改造城市道路24条，加快市区第二水厂、海原第二污水处理厂等项目建设。实施 “大河之舞”二期工程，规划建设环城森林公园，做精城市绿化、亮化、美化，打造“一街一品” “一街一景”，加快建设生态宜居城市。加快推进城市“六创”，建成政务、生活、旅游等综合性服务呼叫平台，建设“智慧城市”。扎实推进海原大县城建设和中宁县城新区建设，引导沙坡头区与中宁县相向发展，不断提升沿黄城市的聚集承载力。

让农村更加美丽。依托清水河城镇产业带，编制完成《中卫城乡一体化发展规划》和《新农村规划》，实施美丽城镇和美丽村庄建设工程，打造沙坡头区宣和、中宁县大战场、海原县七营等9个特色小城镇和东园镇史湖村等15个美丽村庄，改造农村危房5000户，逐步形成布局合理、特色鲜明、风格迥异的村镇体系。实施农村环境卫生综合整治，配套完善农村环卫设施，农村垃圾全部实现集中收集、集中转运、集中处理，切实改善农村人居环境。

让环境更加宜人。实施美丽中卫建设行动计划，加快防沙治沙、南华山生态修复及水源地涵养林、移民迁出区生态修复工程建设，完成绿化造林22.5万亩。出台环境保护执法管理办法等10项环保制度，实行重点企业对标管理，33家化工企业环境在线监测，坚决淘汰一批高耗能、高污染企业生产线。采取第三方运营模式，提标改造中卫工业园区污水处理厂，对化工废水进行集中专业化处理，实现废水全部达标排放。实施大气环境数据实时监测和公开发布，建立健全查处环境违法行为联动机制。完善专家评审和公众参与评审相结合的环境影响评价机制，积极开展重点行业企业清洁生产强制性审核。制定项目准入清单，凡国家产业目录限制和禁止类项目坚决不审批，对不符合国家环保政策的项目不立项，为子孙后代保护好蓝天白云、绿水青山。

五、不断深化改革开放，切实优化发展环境

实施改革创新行动计划，研究制订一系列好政策、好机制、好制度，打造充满活力、具有巨大吸引力、能够不断产生和造就千千万万创业者的区域优势。

加快行政体制改革。充分发挥市场在资源配置中的决定性作用，进一步释放市场活力。继续深化行政审批制度改革，进一步简政放权，强化沙坡头区、海兴开发区、沙坡头旅游经济开发试验区服务职能，实现“两优”行政审批制度常态化。大力推行网上审批、“一门受理、内部流转、多证联办”等审批服务模式，切实提高服务质量。建立非公经济服务中心，积极为企业提供保姆式服务。实行严格的追责问责制度，对破坏中卫改革发展的人和事，一追到底，一查到底，全力打造在西部最优、比东部更优的发展环境。

推进重点领域改革。充分发挥规划龙头引领作用，做到经济社会发展规划、城乡建设规划、土地利用总体规划 “三规合一”，引导生产力在国土空间上合理布局。创新工业园区管理模式，合理划分工业园区职能权限。建立社会资本参与基础设施建设新机制。加快电力改革，建立大用户直购电模式，建设中宁工业园区自备电厂和动力电站，健全电力直接交易机制，完成110千伏以上工业企业纳入电力需求管理平台。推进职业教育办学体制改革，探索校企人才互兼互聘机制。深化医药卫生体制改革，加快公立医院改革。探索建立第三方社会组织参与信访矛盾纠纷化解机制。在天然气、供热等领域引入市场定价机制。完成农村土地确权登记，规范农村产权交易行为，实现农村资源效益最大化。

深化财税金融改革。创新投融资体制，积极对接，探索设立中关村产业园产业投资基金、新能源基金、旅游专项基金、枸杞等特色农业基金，完善政府偿贷资金，发挥财政资金杠杆作用。支持建立资本交易中心，搭建融资平台，拓宽融资渠道，支持企业通过债券市场、新三板等资本市场直接融资，促进混合所有制、现代公司制企业发展。推进财政零基预算，强化财政预算约束，加强财政预算执行，建立非公共财政支出项目清单，做到科学理财、依法理财。建立担保体系，探索以土地收益作担保进行融资。创新金融产品、服务方式，促进实体经济发展。引进私募股权基金，支持民营资本成立银行，积极发展村镇银行，推广政府与社会资本合作（PPP）模式。积极发展“三农”金融和普惠金融，促进农村繁荣发展。

促进军民融合产业发展。加强国防动员建设和全民国防教育，拓展双拥共建内容，争创全国双拥模范城市。加强民兵应急队伍建设，提高遂行任务能力。落实新兵征集退伍安置责任机制，营造拥军优属浓厚氛围。坚持任务牵引、项目承载，推进军民融合深度发展。

六、全面推进依法行政，努力建设人民满意政府

深入学习贯彻十八届四中全会精神，认真落实自治区全面推进依法治区和市委依法治市实施意见，实施依法治市行动计划，加快建设法治政府。

坚持科学执政。坚定不移地解放思想，坚决破除阻碍发展的惯性思维和体制机制，以思想大解放推动经济大发展。健全政府依法决策机制，普遍建立法律顾问制度，把公众参与、专家论证、风险评估、合法性审查、集体讨论决定作为重大行政决策的法定程序。在作出重大决策时，主动向党委、人大报告，与政协沟通。建立行政机关内部重大决策合法性审查机制和重大决策终身责任追究制度及责任倒查机制，切实提高政府领导班子科学执政的能力和水平。

坚持依法行政。自觉接受人大及其常委会的法律监督和政协的民主监督，积极推进协商民主制度化建设，广泛接受社会公众和舆论监督。依法转变和规范政府职能，厘清政府、市场、社会的关系，充分发挥市场在资源配置中的决定性作用。进一步简政放权，建立政府部门“权力清单” “负面清单”和“责任清单”，做到法无授权不可为、法无禁止皆可为、法定职责必须为。坚持用法治思维和方式处理行政事务，用法治引领改革发展、推动民生改善、促进社会公正。

坚持务实勤政。巩固党的群众路线教育实践活动成果，切实转变工作作风。开展好群众评议行政机关和干部活动，自觉接受社会监督。强化政府工作目标任务的考核和督查，按照任务项目化、项目目标化、目标节点化、节点责任化的要求，将每项重点工作、重点项目分解落实到人，明确工作标准和完成时限，强化过程管理和绩效考核，做到监督与落实并行、问责与问效同步，真正使干部把心思用在工作上、把功夫下在落实上。

坚持廉洁从政。扎实推进清廉中卫建设，全面落实党风廉政建设“两个责任”，严格落实“八项规定”，树立为民、务实、清廉的良好形象。健全规范和制约权力运行的制度体系，认真落实“一把手”五不直接分管制度，完善土地出让、产权交易、工程招标、政府采购等公共资源交易体系。不断完善以电视、报纸、广播、网络、微博和微信“六位一体”的媒体联动问政机制，加强对重点领域不正之风的专项治理，强化领导干部经济责任审计和行政问责，防止权力失控、决策失误、行为失范。坚持“零容忍”惩治腐败，加大对违法违纪案件的查处力度，努力做到干部清正、政府清廉、政治清明。

雄关漫道真如铁，而今迈步从头越。加快二次创业新征程，推动产业转型升级，打造副中心城市，建设陆上、网上丝绸之路，我们肩负的使命更崇高，承担的任务更繁重，发展的信心更坚定。让我们在自治区党委、政府和市委的坚强领导下，以实打实的作风干工作，以踏石留印、抓铁有痕的劲头抓落实，全面完成“十二五”各项目标任务，为建设开放富裕和谐美丽中卫不懈奋斗！
